# Active search of adult patients with persistently low serum alkaline phosphatase levels for the diagnosis of hypophosphatasia

**DOI:** 10.20945/2359-3997000000347

**Published:** 2021-04-12

**Authors:** Lucio Henrique Rocha Vieira, Kleison Cordeiro Peixoto, Caroline Leal Flósi, Maria Lucia Fleiuss de Farias, Miguel Madeira

**Affiliations:** 1 Universidade Federal do Rio de Janeiro Hospital Universitário Clementino Fraga Filho Divisão de Endocrinologia Rio de Janeiro RJ Brasil Divisão de Endocrinologia, Hospital Universitário Clementino Fraga Filho, Universidade Federal do Rio de Janeiro, Rio de Janeiro, RJ, Brasil; 2 Hospital Universitário Clementino Fraga Filho Laboratório de Bioquímica Rio de Janeiro RJ Brasil Laboratório de Bioquímica, Hospital Universitário Clementino Fraga Filho, Rio de Janeiro, RJ, Brasil

**Keywords:** Alkaline phosphatase, hypophosphatasia, bone

## Abstract

**Objectives:**

Alkaline phosphatase (ALP) is the main laboratory marker of hypophosphatasia (HPP), a rare disease unknown to most physicians. The prevalence of HPP has been widely discussed in the literature due to the diverse phenotypes of HPP. The purpose of this study was to search for patients with hypophosphatasemia based on previous biochemistry tests and reevaluate them to confirm the diagnosis of HPP.

**Subjects and methods:**

A total of 289,247 biochemical tests for ALP in adults were performed from 2015 to 2019 in two tertiary hospitals in Rio de Janeiro were reviewed (Clementino Fraga Filho University Hospital – HUCFF – and Bonsucesso Federal Hospital – BFH).

**Results:**

A total of 1,049 patients were identified with ALP levels below 40 U/L, and 410 patients had hypophosphatasemia confirmed by at least two exams. After the active search of medical reports and/or interviews based on structured questionnaires, 398 subjects were excluded due to secondary causes of reduced ALP. The remaining 12 patients were invited to attend the medical consultation at HUCFF, accompanied by at least one first-degree relative. None of the patients or their relatives had a history or clinical manifestations consistent with HPP. Serum ALP was within reference values in all relatives, but persistently low in further laboratory evaluation in all the 12 patients, in whom secondary causes were ruled out. Thus, we cannot exclude the possibility that they might carry the mutations associated with HPP.

**Conclusion:**

Further image evaluations and genetic testing would be appropriate to confirm this asymptomatic adult form of HPP.

## IINTRODUCTION

Serum alkaline phosphatase (ALP) is one of the main tests requested in clinical practice, both in outpatients as well as in hospitalized patients. A low total ALP level is the main biochemical marker of hypophosphatasia (HPP). HPP is an autosomal dominant or autosomal recessive inborn error of metabolism with an extraordinary range of severity, and HPP is particularly caused by loss-of-function mutations within the gene that encodes the tissue-nonspecific isoenzyme of alkaline phosphatase (TNSALP) (1). There are North American, European and Canadian publications that estimate the prevalence of severe cases as 1/100,000 to 1/297,000 born alive (2,3).

The main problem in routine practice is that only high values of ALP are usually considered, as they may be associated with liver or bone diseases. In fact, low values should also be taken into account, mostly in patients with bone complaints. The identification of persistent hypophosphatasemia – which is a laboratory abnormality, not a disease – is very important because it may be an indicator of an asymptomatic adult or a carrier of a recessive mutation. Limited evidence exists regarding the features that signal a potential association between hypophosphatasemia and HPP in adults (4).

Although HPP is a very rare disease, bone and dental disturbances are well documented in the literature in all classifications of the disease, since ALP is an important enzyme responsible for bone and teeth formation (5). The recognition of the disease in asymptomatic or oligosymptomatic elderly people with osteoporosis or fragility fractures would prevent the disastrous use of bisphosphonates, which is associated with worsening of bone mineralization (6). On the other hand, in young patients in the reproductive phase, genetic counseling would be important if hereditable recessive forms are identified (7). To date, there are no studies in Brazil regarding persistent hypophosphatasemia and its manifestations in adults. The main goal of this study was to identify patients with persistently low levels of ALP, after excluding secondary causes, in two tertiary hospitals in Brazil. As a secondary goal, we aimed to investigate family history, identifying ALP level reductions or possible signs and symptoms of HPP.

## MATERIALS AND METHODS

A total of 289,247 biochemical tests of ALP were performed in adults (>18 years old) from January 2015 to December 2019 (n = 249,216) at the Clementino Fraga Filho Hospital (HUCFF) and from January to December 2015 (n = 30,031) at Bonsucesso Federal Hospital (BFH) were evaluated. The study was approved by the Reserch Ethics Committee of BFH and registered on “Plataforma Brasil” (CAAE number: 11466919.3.0000.5253). The screening led to 1,049 patients with at least one ALP result below 40 IU/l [cut-off point for adults according to most publications on this topic (8)], of whom 410 had at least two exams showing persistently low ALP levels at intervals of more than 30 days. The study evaluated all hospital specialties divisions, except the pediatrics department.

Data collection was performed through an active search and study of medical files as well as via telephone contact with patients and family members and interviews with patients with persistently low ALP levels. After the active search, 398 patients were excluded due to secondary causes of hypophosphatasemia. The ALP fluctuations (74%) could be justified by some diseases that can modify the bone turnover or may hinder the interpretation of ALP activity (pre-analytical or analytical interference). The remaining 12 patients (2.92% of 410 patients with at least two results < 40 U/L) were invited to come to HUCFF with their first-degree relatives for a clinical and laboratory study. All of them were subjected to investigative anamnesis addressing the signs and symptoms of HPP, such as bone abnormalities, early deciduous tooth loss, and family history of bone abnormalities based on a specific questionnaire, and the patients had blood samples drawn for repeat ALP examination by other biochemical methods.

During these five years (2015 to 2019), at least three different types of equipment for laboratory tests had been used by these hospitals. Tests for ALP were performed using Beckman Coulter model AU 5.800, Siemens dimension RXL, and Wiener BT-3000 equipment. The enzymatic method used to assess the activity consists of the colorimetric method recommended by the International Federation for Clinical Chemistry (IFCC) (9).

## RESULTS

**Figure 1:**
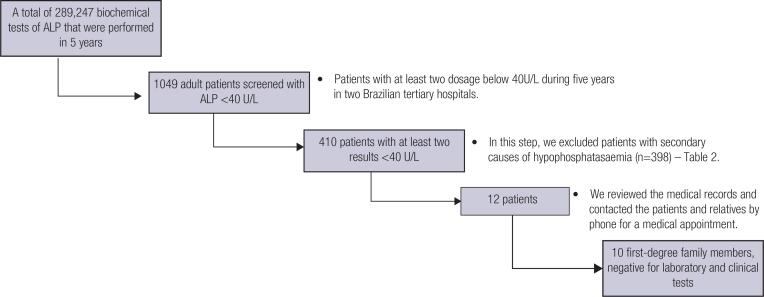
Study flowchart – patient selection.

Our screening identified 12 adult patients with hypophosphatasemia, without any reason that could justify this laboratory alteration (Figure 1). The patients ranged in age from 23 to 70 years (median 43 years), three males and nine females. None reported any skeletal complaints, such as bone pain, childhood deformities or fragility fractures, but one woman complained of spontaneous muscle pain. They also did not report having chondrocalcinosis, nephrolithiasis, nephrocalcinosis, or early dental loss.

In male patients, ALP levels varied from 25 to 38 U/L (median 37 U/L), and in female patients, ALP levels varied from 18 to 37 U/L (median 32.5 U/L). We evaluated at least one first-degree family member of ten patients, and we did not find persistently low levels of ALP or clinical symptoms that suggested bone disease or early deciduous tooth loss. Only two patients did not have their families evaluated, both due to social issues, but there were no reports of bone deformities, and there were no health concerns. The relatives of ten patients were tested: children (n = 8), a mother (n = 1), and a sister (n = 1); however, all relative had normal ALP levels, as shown in Table 1.

**Table 1:** Clinical characteristics of patients with persistently low alkaline phosphatase levels

Patient	Age	Sex	Main diagnosis	Low ALP levels confirmed by three different methods/lower and higher values	Bone disease or fracture	Early tooth loss	Muscle complaints	Lithiasis, extraskeletal calcification	Number of investigated relatives affected/ yes or no
1	50	F	Cured hepatitis B	Yes/23-27	No	No	No	No	1/no
2	49	M	Hypertension Cured hepatitis C	Yes/25-33	No	No	No	No	0
3	38	F	Grade 3 obesity	Yes/18-29	No	No	No	No	1/No
4	39	F	Deep vein thrombosis	Yes/35-37	No	No	No	No	1/No
5	37	F	Grade 2 obesity	Yes/28-36	No	No	No	No	1/No
6	43	F	Subclinical hypothyroidism	Yes/32-33	No	No	No	No	1/No
7	43	M	Grade 1 obesity	Yes/28-30	No	No	No	No	1/No
8	46	F	Diabetes mellitus and hypertension	Yes/34-36	No	No	Yes	No	1/No
9	70	F	Resistant hypertension	Yes/27-37	No	No	No	No	1/No
10	44	F	Overweight	Yes/35-36	No	No	No	No	1/No
11	23	M	Low weight	Yes/25-38	No	No	No	No	0
12	32	F	Late postoperative cholecystectomy	Yes/30-33	No	No	No	No	1/No

ALP: alkaline phosphatase.

The main causes of exclusion were fluctuations in ALP levels (74%). Despite the initial screening, we observed that subsequent tests results demonstrated in the medical files showed values higher than 40 IU/l during the five years of the retrospective study. The other causes of exclusion have been well described in the literature (10), such as the use of glucocorticoids, chemotherapy medication in oncologic patients, immunosuppressants, fibrates, and bisphosphonates, cleidocranial dysplasia and Mseleni joint disease (Table 2).

**Table 2:** Causes for patients exclusion

Reason for exclusion	Number of patients excluded	Percentage of patients excluded
Cardiac bypass	2	0.5%
Fibrates	5	1.25%
Multiple myeloma	2	0.5%
Malnutrition	1	0.25%
Cancer	21	5.27%
Glucocorticoids	36	9.04%
Chemotherapy	14	3.51%
Immunosuppressants	17	4.27%
Bisphosphonates	4	0.50%
Fluctuations in ALP levels	296	74%
Total	398	100%

ALP: alkaline phosphatase.

## DISCUSSION

HPP is a rare disease (RD) caused by a mutation in the *TNSALP* gene, which codifies non-tissue-specific ALP. It may present with recessive or dominant autosomal transmission, and more than 300 mutations have been identified (8). Patients with HPP have decreased serum ALP levels and elevations of the natural substrates pyridoxal-5’-phosphate (vitamin B6) and phosphoethanolamine. However, only the level of ALP is required for the diagnosis of HPP (10).

It was described in a previous study that 50% of adults with repeatedly low ALP levels had a mutation in the *TNSALP* gene (11). All mutations were predicted to impair the enzyme activity. In fact, haploinsufficiency was enough to reduce serum ALP activity, and in approximately one-half of the patients with identified mutations, there was biochemical evidence of the accumulation of phosphorylated substrates. Therefore, these adults may carry pathogenic, benign, or uncertain mutations that cause genetic variations. There is a positive correlation between the types of pathogenic mutations and high levels of substrates in blood and urine (8).

Despite its rarity, HPP should be fundamentally recognized in patients with persistently low ALP levels. Adult HPP presents after the age of 18 years and can include dentition loss, fractures, pain, and disability (12). The condition should be especially suspected in patients with recurrent metatarsal fractures that can heal poorly (13). Theoretically, dental anomalies may exist in every subtype of HPP (14). Comorbidities, such as renal failure, can affect the phenotype of HPP (15).

We detected 12 adult patients with this biochemistry profile who had no manifestations of HPP. These 12 patients may be within the spectrum of the adult form of HPP, which is generally oligosymptomatic or asymptomatic. ALP levels were normal in the 10 relatives tested, who were also completely asymptomatic. The genetic test can be used to confirm mutations associated with HPP, and this confirmation would allow for genetic counseling, as well as choosing adequate alternatives for osteoporosis or fracture treatment in adults, avoiding bisphosphonates (16). The molecules of these medications are very similar to one of the substrates that accumulates due to low ALP activity (inorganic pyrophosphate, an inhibitor of bone formation). Thus, the risk of excessive inhibition of bone turnover may lead to bone fragility and rare side effects such as atypical femoral fractures (11).

In the literature, the prevalence of persistently low ALP levels was 0.06% in the study of a large rural American population (17) (n = 885,165), constituted predominantly by individuals of northern European descent with no relevant ethnic diversity, with a mean age varying from 46 to 55 years. In this study, an important sex effect was observed, in which more women (n = 171; 64%) presented with low ALP levels than men (n = 98; 36%). These patients had more radiographic evidence of chondrocalcinosis, calcific periarthritis, enthesopathy, and diffuse idiopathic skeletal hyperostosis than the general adult patient population (p < 0.001). The exclusion criteria were the same as those used in the present study.

From a different perspective, a study conducted in a French tertiary hospital with fewer patients (2) (n = 48,755) showed the prevalence of persistently low ALP levels to be 0.13%. This study selected patients, especially from the departments of rheumatology, endocrinology, internal medicine and gastroenterology, but excluded patients from the emergency room. The mean age varied from 46.5 ± 17.7 years of age, and 73% were females, a gender predominance also found in our cohort and in other studies (2,17). Whether this predominance of females in the adult form of HPP is really true, more scientific researches are still needed to confirm. These patients showed radiographic evidence of chondrocalcinosis, a previous history of fractures and rickets in childhood, in addition to a healing delay of fractures.

One of the challenges of our study was to find the prevalence of HPP in our adult Brazilian population, because even in two tertiary healthy service no one severe case was identified. We highlight the fact that the population studied is very miscegenated, all from one state, with low socioeconomic levels, and with many undertreated comorbidities, which can delay the diagnosis of this rare disease. After our retrospective cohort study, we can hypothesize that the prevalence of syntomatic adult forms of HPP in Brazil may be lower than described in the literature. We can also speculate that this strategy was not good enough to identify potential patients, or that we would need to increase our sample size. Another possible explanation is that more severe forms in childhood can prevent individuals to reach the adult age due to high mortality.

Some mutations in the Brazilian population have been described. A 36-year-old male presented with multiple fractures, short stature, and early craniosyostosis. His level of ALP was very low (6U/L). Genetic testing showed a homozygous missense mutation in *ALPL* gene c.443 C>T: p.Thr148Ile, also identified in his mother (18). Another case of a Brazilian child whose parents and sister had reduced ALP and increased PLP levels was published. His mother (36-year-old) was diagnosed with rickets in her childhood while his father had no history of fractures bone deformity or other clinical abnormalities observed. A homozygous c.98C>T (p.Ala33Val) missense mutation in the *TNSALP* gene was identified (19).

As mentioned above, HPP is a rare disease (RD). Currently, there are more than 6,000 RDs known in the world, of which 71.9% are of genetic origin (8), such as HPP. RDs are heterogeneous, numerous, and geographically very disparate. Few are curable, most are chronic, and many result in premature death as one of the possible manifestations of HPP. The challenges resulting from the low prevalence of RDs are knowledge scarcity and the chronic and life-threatening nature of RDs (20). Increasing numbers of patients and relatives are becoming involved in the management of their condition through the use of digital platforms, social media and tools to enable and increase their confidence as well as through the empowerment of families and the fundraising of research projects. The main responsibility lies with health professionals, who must keep themselves informed and updated to come to a correct diagnosis and treatment of these diseases.

The major limitation of the present study was not to further evaluate each patient of these 12 patients regarding some aspects like genetic testing, bone density and microarchitecture, and imaging diagnosis of chondrocalcinosis, nephrolithiasis and nephrocalcinosis. On the other hand, the great relevance of this study is to raise awareness among the medical community concerning a rare disease with a prevalence and an incidence not yet largely studied in the Brazilian population. Patients who have persistently low ALP levels could be recognized potentially as having an RD. These patients can be carriers of autosomal or recessive genetic mutations with low penetrance, and there is a risk that they could transmit this severe disease to their descendants. This important information enables genetic counseling and the prevention of a potentially catastrophic disease for family members.

Finally, we highlight the low prevalence of primary persistent hypophosphatasaemia in our sample and that our patients with persistently low ALP levels did not present any clinical manifestations of HPP, maybe because the adult form of HPP is generally less severe than the infantile types. Further studies are needed to confirm this prevalence and to investigate these asymptomatic patients in relation to image evaluations and genetic testing.
